# Comparing the Effects of Different Injection Techniques Used in Lip Augmentation on Filler Migration and Patient Satisfaction

**DOI:** 10.7759/cureus.64716

**Published:** 2024-07-17

**Authors:** Omer Buhsem

**Affiliations:** 1 Plastic Surgery, Private Practice, Bursa, TUR

**Keywords:** facial contour, vermilion border, filler injections, filler migration, lip rejuvenation

## Abstract

Objective: Lip rejuvenation has several aims, including enhancing lip volume, adjusting the upper and lower lip’s length, diminishing fine lines, contouring and redefining the cupid bow and vermilion border, and eversion of the vermilion. Within the scope of this research, we aimed to compare popular injection techniques to augment the size of the lips.

Materials and methods: This randomized retrospective study included 216 female patients aged 19 to 39, who desired a lip filler treatment from 2017 to 2023. Pre- and post-procedure results were elaborated with top-to-the-bottom technique in Group 1, bottom-to-the-top technique in Group 2*,* and lateral-to-medial techniques in Groups 3 and 4.Once the patients were sufficiently anesthetized, the hyaluronic acid at a concentration of 20 mg/mL with 0.3% (3 mg/mL) lidocaine, was used in all groups. Patients were followed up for three weeks. Patient satisfaction scores were evaluated on a scale from 0 to 5 using a survey on the last follow-up day.

Results: A statistically significant difference was found between the groups regarding satisfaction scores (p<0.05). The patient satisfaction scores after injection were 4.78/5 in Group 1, 3.70/5 in Group 2, 4.15/5 in Group 3, and 3.85/5 in Group 4. Kruskal-Wallis variance analysis for more than two groups revealed statistically significant differences between Group 1 and Group 2 (p<0.001), Group 1 and Group 3 (p<0.001), Group 1 and Group 4 (p<0.001), and Group 2 and Group 3 (p=0.009) (Mann Whitney U-Test with Bonferroni adjusted). No major complication was observed in any of the patients.

Conclusion: In this study, patient satisfaction was found to be highest in the group with needle orientation top to bottom, taking into account migration to the upper lip. These findings showed that the direction of the needle during injection also determines the direction of distribution of the filler on the lip and may be an important factor in patient satisfaction.

## Introduction

During the natural aging process of our skin, there is a decrease in subcutaneous adipose tissue and dermal support, the skin becomes thinner and loses its elasticity. Distortions appear in the facial appearance; cheeks and lips begin to lose their fullness; deep wrinkles, prominence of nasolabial folds, and other undesirable skin defects may occur due to soft tissue atrophy as well as skeletal changes [1]. Different techniques can be used for lip augmentation such as dermal fillers and dermofat grafts [2]. Dermal filler injections are an alternative for those who do not want aesthetic surgery. In some cases, the filling can be applied to delay surgical operations or as an adjunct treatment to surgery [3].

Dermal filler injections are used to place various fillers into the dermis or subcutaneous tissues. Fillers are used for volume replacement and enhancement procedures. These substances are not autologous but synthetic and they can reduce wrinkles, correct dermal depressions, and scars caused by trauma, enhance facial contours, and moisturize the skin [4].

Some important factors affect the cosmetic result in the filling process. These factors are the selection of the ideal product according to the localization and the depth to be injected, the injection technique used, the appropriate patient selection, the patient's expectations, the experience of the physician, and the use of botulinum toxin injection, laser, and chemical peeling [5].

The appropriate product should be selected according to the area of planned use. Features of the skin of the region, vascularization, thickness, tension, mechanical movements, tissue surface, line, and wrinkle depth are important. High-viscosity products with thick needles (27G) are an appropriate choice for a large atrophic scar or nasolabial sulcus, also where bulk or volume augmentation is needed such as cheeks, jawline, etc. [6]. In the correction of thin, superficial lines such as around the eyes and the mouth, products with high fluidity should be injected intradermally with fine-tipped needles (30G-33G). Fillers made superficially may appear as a color change on the surface, while those made too deep can be lost in the tissue [7].

The patient's age, lifestyle, habits (smoking, diet, exercise), expectations, skin type and elasticity, and medical history (allergy history, medications used, previous fillers) should be well evaluated. The expected improvement in the result of the procedure should be well explained. Contour disorder or elasticity of the skin can be evaluated with the “stretching test.” The best results are obtained if the wrinkle disappears when the area to be treated is stretched between two fingers [8].

Using fillers to enhance lip volume is now one of the most common aesthetic procedures. Lip rejuvenation has several aims, including enhancing lip volume, adjusting the length of the upper and lower lip, diminishing fine lines, contouring and redefining the cupid bow and vermilion border, and eversion of the vermilion. Having a comprehensive understanding of injection techniques is important, which is as important as the filler material [9].

There are a growing number of techniques available in the literature. However, as the types of filler materials, application techniques, practitioners, and the number of procedures increase, many minor and major complications have been described in the literature related to lip fillers. The possible undesired results or complications that we do not particularly want on the lips are excessive fullness, filler migration, shapeless lips, asymmetry, and lumps [10].

In this study, we aimed to compare different injection techniques to augment the size of the lips. The outcomes of four filler injection techniques with different needle directions applied to the upper lip were compared. This study was conducted to determine whether the direction of the needle during injection also affects the direction of distribution of the filler on the lip and whether it has a role in filler migration to the upper lip. To our knowledge, there are not enough studies in the literature comparing these techniques and their results.

## Materials and methods

This retrospective study based on patient records included 216 female patients aged 19 to 39, who desired a lip filler treatment from 2017 to 2023 and were injected by the same surgeon with four different techniques. All procedures followed were in accordance with the ethical standards of the responsible committee on human experimentation (institutional and national) and with the Helsinki Declaration of 1975, as revised in 2008. Ethics committee approval was granted from Necmettin Erbakan University, Konya, Turkey on June 2, 2023 with protocol number 4360, and informed consent was obtained from all participants.

Both medical and dental history were taken for all patients, as well as the history of any previous injectables. Healthy Caucasian women 19 years or older who had not received any previous filler injections in the last year were included in the study. Subjects were excluded if they had any conditions contraindicating hyaluronic acid filler or underwent previous treatments that could impact the effect of the filler. Those who had lip fillers in the last two years, those who had lip surgery, extremely thin lips, had any perioral infections in the last six months or frequent lip herpes infections (more than twice a year), and heavy smokers (those who smoke ⩾25 or more cigarettes a day), were excluded from the study.

Injection techniques

**Figure 1:**
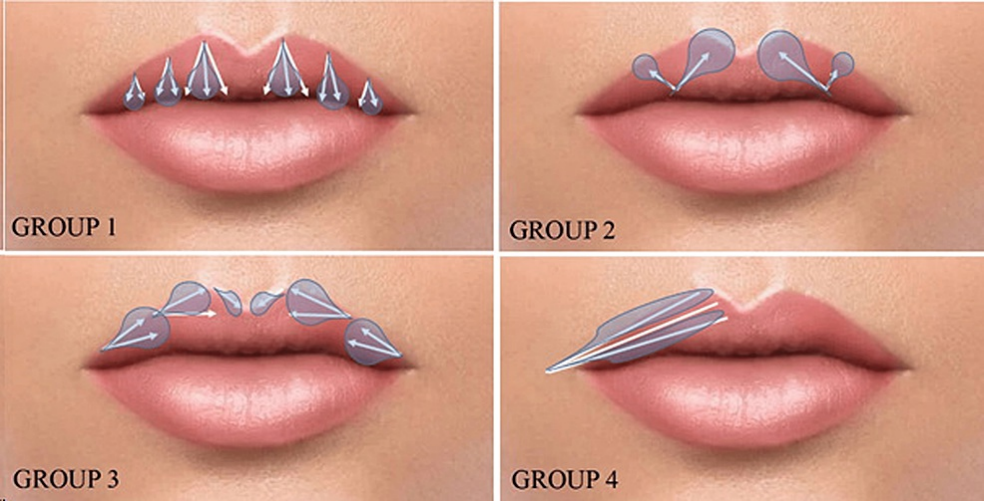
Top-to-the-bottom technique in Group 1, bottom-to-the-top technique in Group 2, and lateral-to-medial techniques in Groups 3 and 4. The direction of the needle also determines the distribution of the filler.

Full assessments were performed for the mouth at rest and dynamics, any asymmetry, skin quality, lip ratio, and shape. The patients were selected from medical records as four different groups based on the direction of the injected needle and cannula. Group 1 included the top-to-the-bottom technique with a needle, Group 2 bottom-to-the-top technique with a needle, Group 3 lateral-to-medial technique with a needle, and Group 4 lateral-to-medial technique with a cannula (Figure 1). The perioral area was cleansed with the antiseptic octenidine dihydrochloride. Block anesthesia was performed intraorally with a 2 cc prilocaine hydrochloride injection (Citanest) in all groups. Submucosal anesthetic multıple injections into the upper and lower mucobuccal fold for block anesthesia were preferred because it does not distort the lip shape, do not cause swelling like topical anesthetic creams, and the injection is more comfortable for the patient. Patients were warned to avoid hot drinks after anesthesia until the effect subsided.

Once the patients were sufficiently anesthetized, the hyaluronic acid at a concentration of 20 mg/mL with 0.3% (3 mg/mL) lidocaine (YVOIRE Volume Plus®, LG Chem, Seoul, South Korea), that raw hyaluronic acid material approved by the U.S. Food and Drug Administration (FDA), was used in all groups. All patients received a filling application of 0.6 ml to the upper lip. The same plastic surgeon performed the application using the same injection plane (submucosal 2 mm depth) in each group. A standard 30-gauge needle was used in Groups 1, 2, and 3. A 23-gauge cannula was used in Group 4. A different injection technique was used in each group. Needle direction was top-to-bottom in Group 1, bottom-to-top in Group 2, lateral-to-medial in Group 3, and cannula direction was lateral-to-medial in Group 4 (Figure 1). All injections were done using the linear threading method. The beveling of the needle (facing upwards to the mucosa at a 30-degree angle) was the same in each group. It was administered in the clinical setting in accordance with the standard operating procedure for the administration. Patients were followed up for three weeks. Patient satisfaction scores were assessed on a scale of 0 to 5 (0: very dissatisfied; 5: very satisfied) using a questionnaire applied by a blinded evaluator medical aesthetic doctor on the last follow-up day, taking into account filler migration to the upper lip.

Statistical analysis

Data were analyzed using the IBM SPSS Statistics for Windows, Version 23 (IBM Corp., Armonk, NY, USA). Characteristics of patients, such as n (percentage) or mean, standard deviation (SD), and median (minimum-maximum) for categorical and continuous variables, respectively, were reported. The continuous variables were compared among groups using Kruskal-Wallis tests. The statistical significance of differences between groups was determined by Kruskal-Wallis, followed by post hoc Mann Whitney U-Test Bonferroni adjusted. The p-value was set at <0.05 for statistical significance.

## Results

A total of 216 patients were included within the scope of this research (Table 1). All patients had successful outcomes.

**Table 1:** Patients and details of each group.

	No. of patients (n=216)	Injection tool and size	Injection area	Injection volume	Needle direction
Group 1	54	30-gauge needle	Upper lip	0.6 ml	Top to bottom
Group 2	54	30-gauge needle	Upper lip	0.6 ml	Bottom to top
Group 3	54	30-gauge needle	Upper lip	0.6 ml	Lateral to medial
Group 4	54	23-gauge cannula	Upper lip	0.6 ml	Lateral to medial

Patients were evaluated 21 days after injection and patient satisfaction scores were assessed by a medical aesthetic blind evaluator doctor on a scale from 0 to 5 using a survey (0: very dissatisfied; 5: very satisfied) (Table 2). No major complication was observed in the three weeks follow-up in any of the patients.

**Table 2:** Distribution of patients according to their satisfaction scores in each group.

	Group 1 (n=54)	Group 2 (n=54)	Group 3 (n=54)	Group 4 (n=54)
	n (%)	n (%)	n (%)	n (%)
Very dissatisfied	0 (0.0)	0 (0.0)	0 (0.0)	0 (0.0)
Somewhat dissatisfied	0 (0.0)	0 (0.0)	0 (0.0)	0 (0.0)
Neither satisfied nor dissatisfied	1 (1.9)	21 (38.9)	7 (13.0)	14 (25.9)
Somewhat satisfied	10 (18.5)	28 (51.9)	32 (59.3)	34 (63.0)
Very satisfied	43 (79.6)	5 (9.3)	15 (27.8)	6 (11.1)

Patient satisfaction scores after injection were 4.78/5 in Group 1, 3.70/5 in Group 2, 4.15/5 in Group 3, and 3.85/5 in Group 4 (Table 3). When the table was examined, a statistically significant difference was found between the groups regarding satisfaction scores (p<0.05). As a result of the Mann-Whitney U-Test with Bonferroni correction, it was determined that the difference was caused by the differences between Group 1 and Group 2, Group 1 and Group 3, Group 1 and Group 4, and Group 2 and Group 3. Kruskal-Wallis variance analysis for more than two groups revealed statistically significant differences between Group 1 and Group 2 (p<0.001), Group 1 and Group 3 (p<0.001), Group 1 and Group 4 (p<0.001), Group 2 and Group 3 (p=0.009) (Mann-Whitney U-Test with Bonferroni adjusted) (Table 3).

**Table 3:** Comparison of scores in treatment groups. A statistically significant difference was found between the groups regarding satisfaction scores (p<0.05). p<0.05 significant; Kruskal Wallis variance analysis for more than two groups; differences between Group 1 and Group 2 (p<0.001), Group 1 and Group 3 (p<0.001), Group 1 and Group 4 (p<0.001), Group 2 and Group 3 (p=0.009) according to score is significant p<0.05; Mann Whitney U-Test with Bonferroni adjusted.

Treatment groups	Score		p-value
	Mean ± SD	Median (Min-Max)	
Group 1	4.78 ± 0.46	5 (3-5)	<0.001
Group 2	3.70 ± 0.63	4 (3-5)	
Group 3	4.15 ± 0.63	4 (3-5)	
Group 4	3.85 ± 0.60	4 (3-5)	

## Discussion

Lip rejuvenation has three important areas; the vermilion border, the red lip region, and the philtrum. The ideal vermillion height of the upper to lower lip has been described using the “golden ratio” of 1:1.618 [10]. The filler is injected into the submucosa for volume storage in the lip. Excessive volume can cause a duck lip appearance, and fine lip movements can be lost. For the lip contour, fillers are given more superficially. Application to the vermilion border elevates the upper and lower lip corners [11]. After the application, the filler can be distributed and molded homogeneously by massage. The application is less painful with fillers containing lidocaine; a painless application can be made by nerve block anesthesia. Excessive volume distribution and deep dermal application should be avoided [12].

Lip augmentation with improved techniques has gained popularity because full lips are often associated with beauty and youth. The importance of the lip in overall facial aesthetics has an impact on positive self-image and self-confidence. In general practice, there is a high demand for lip fillers, especially in the upper lip, by patients. In this regard, this procedure has some desired and undesirable outcomes [13]. A lip augmentation procedure aims to create smooth lips with adequate volume, a well-defined vermilion border, and Cupid’s bow. However, one of the most important undesirable situations is the migration of the filler on the cutaneous part of the upper lip due to the movement of orbicularis oris [14]. Muscle activity of the orbicularis oris muscle complex causes continuous stretching and compression of the lips, and the physical shear forces [15]. Musicians who play instruments such as flutes are not suitable patients, as they may impair lip movements. High amounts may affect lip movements, eating, and drinking [16].

Various injection techniques have been described for lip augmentation. Needle injections are recommended for superficial administration to avoid vascular injury. However, cannulas with blunt ends provide less risk of vascular occlusion, according to anatomical evidence-based research [17]. Nevertheless, small-borne cannulas and using too much force during the injection may violate a blood vessel with subsequent intraluminal filler placement, causing vascular occlusion. On the other hand, recent evidence favors a needle over a cannula, and using a needle instead of a cannula helps the injector become familiar with the most precise placement of the filler [18]. Cannula techniques do not provide much artistry in reshaping the lips; therefore, most clinicians use the needle alone to fulfill this purpose. Some popular techniques, such as Russian lips, require needle injections [19].

This study aimed to evaluate the effect of different needle vector directions (top-to-bottom, bottom-to-top, and lateral-to-medial) on the outcomes of lip filler injections. The primary outcomes assessed were filler migration to the upper lip and overall patient satisfaction. Our findings underscore the significant impact of needle direction on achieving optimal aesthetic results and ensuring patient satisfaction in lip augmentation procedures.

**Figure 2:**
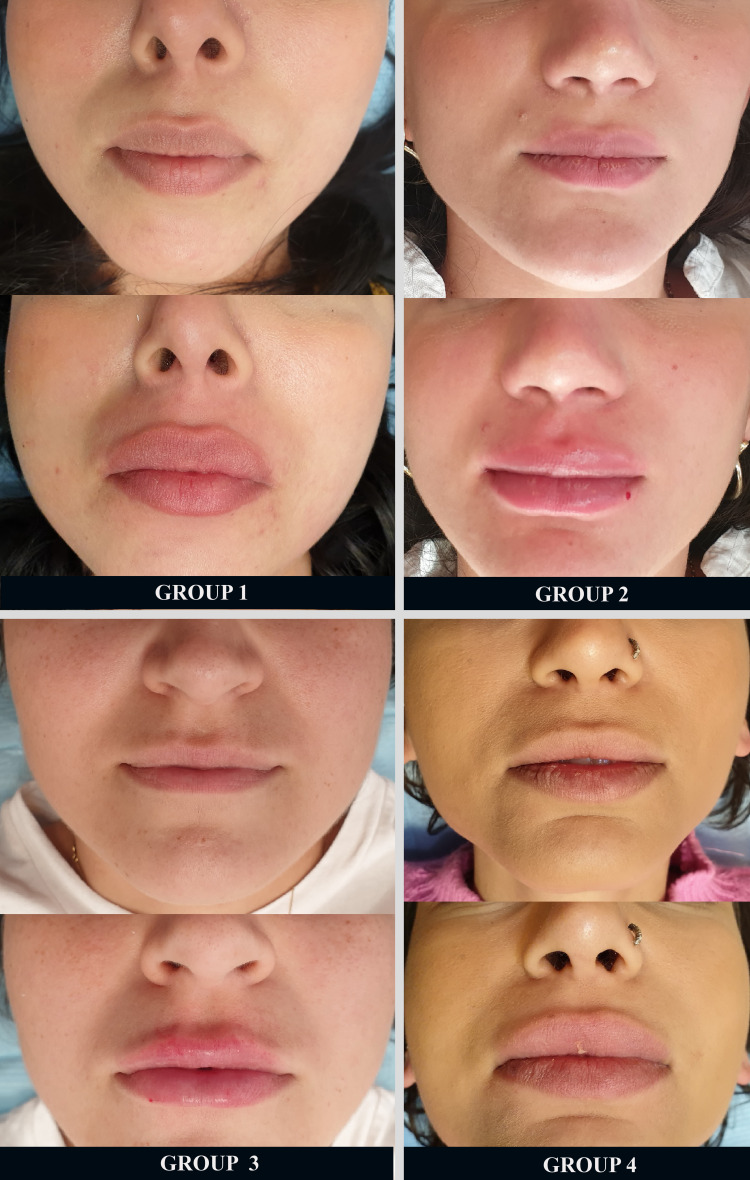
Four different injection techniques used in upper lip augmentation and appearance immediately after injections. Needle direction was top-to-bottom in Group 1, bottom-to-top in Group 2, lateral-to-medial in Group 3, and cannula direction was lateral-to-medial in Group 4.

Lip filler migration, where the injected filler moves from its intended location to adjacent areas, is a concern in aesthetic medicine. Understanding the underlying causes of filler migration can help practitioners adopt strategies to minimize this complication and improve patient outcomes. The primary reasons for lip filler migration include filler type, poor injection technique, patient anatomy, external factors, increased muscle activity, excessive massaging after filler injection, lymphatic spread, and intravascular injection [20]. In literature regarding the injection technique, it has been shown that the result may be affected if the filler is injected into the orbicularis oris muscle, which may be related to some extent to whether a needle or cannula was used [21]. However, there is no data on the direction of the injection that may impact the possible filler migration event. In this study, the group (Group 1) without filler migration to the upper lip provided the best eversion and had the highest satisfaction rate (Figure 2). The injection conducted in Group 1 was the top-to-bottom injection technique.

The physicochemical properties of the filler significantly influence its stability and tendency to migrate. High-viscosity fillers are thicker and more stable, making them less prone to migration compared to low-viscosity fillers. Low-viscosity fillers are more fluid and can move more easily within the tissue [22].

Fillers with high cohesiveness maintain their shape and position better within the tissue, reducing the risk of migration. Cohesive fillers are less likely to spread beyond the injection site, maintaining a more localized effect [23].

The technique used during the injection process is crucial in determining the distribution and stability of the filler. Linear retrograde or anterograde threading technique involves the continuous injection of filler as the needle is withdrawn. If not performed with precision, it can lead to an uneven distribution of filler and increase the risk of migration, particularly if the filler is injected too superficially or too deeply [23]. The linear retrograde threading method was used in all groups in our study.

Individual anatomical differences, such as lip size, shape, and tissue composition, play a significant role in filler migration. Patients with less firm lip tissue may be more prone to filler migration. Softer tissues provide less structural support to hold the filler in place, allowing it to move more easily [24]. Frequent and extensive lip movements, such as those from talking, eating, or facial expressions, can contribute to filler displacement over time. The mechanical forces exerted on the lips can push the filler from its original location [23].

External factors, including post-procedure care and lifestyle habits, can also influence filler migration. Improper care following the injection, such as excessive massaging of the lips or exposure to high heat (e.g., saunas), can lead to filler migration. Patients should follow post-procedure guidelines to ensure the filler remains in place during the initial healing period [25].

Lifestyle habits such as smoking or using straws can exert additional pressure on the lips, contributing to filler migration. Educating patients about the impact of these habits can help in preventing complications [24].

The direction of the needle vector and angle during lip filler injections may play a critical role in determining the distribution and migration of the filler material [24]. Injections performed with an angled vector, particularly those directed at a 45-degree angle relative to the lip contour, showed significantly reduced migration rates. This technique appears to facilitate a more controlled and targeted distribution of the filler, maintaining its placement within the intended areas. These findings are consistent with the principles of tissue mechanics, where angled injections are less likely to breach the natural barriers within the lip structure [22]. Furthermore, the reduced incidence of complications, such as filler migration, in the angled injection group contributed to higher satisfaction scores. Complications like asymmetry or unnatural lip contour not only impact the visual outcomes but also affect the psychological well-being of patients, leading to dissatisfaction [23]. Therefore, the technique employed by the practitioner is crucial in minimizing adverse effects and enhancing patient satisfaction.

While our study provides valuable insights, it is not without limitations. The sample size was relatively small, and the follow-up period was limited, which may impact the generalizability of the results. Future studies with larger cohorts and longer follow-up periods are necessary to validate these findings and explore the long-term effects of needle vector direction on filler migration and patient satisfaction.

Additionally, the role of other factors, such as filler type, injection volume, and individual patient anatomy, should be investigated to develop a comprehensive understanding of the optimal injection techniques. Combining objective assessments with subjective patient feedback will further enhance the quality and applicability of research in this field.

Although filler products are generally very safe, various side effects and complications such as bruising, swelling, redness, tenderness, lumps, allergic reactions, infection, skin necrosis, asymmetry, filler migration, scarring, and vision problems can occur [23]. These complications encountered in the application of fillers can be classified in various ways according to the severity of the clinical picture (mild, serious), the time of occurrence (early period, late period), and their causes [26].

It is normal for temporary edema to occur immediately after the application, which happens with almost all fillers. It occurs immediately after administration, depending on the injection volume and technique. Treatment and precautions are the same as for bruising, eliminating alcohol, avoiding touching, rubbing, or squeezing the treated areas, avoiding exercise, sun, and/or heat, and applying ice or cold packs to the affected areas during and after treatment. Edema due to postinjection trauma is expected to resolve within one week [27]. There is a risk of ecchymosis in all dermal fillers. Collagen-containing fillers cause less ecchymosis than hyaluronic acid, as they stimulate platelet activation. After application, especially in dark-skinned people, post-inflammatory hyperpigmentation (PIH) may develop [28].

Seeing slight erythema on the skin is normal immediately after the injection. If the erythema persists for over five days, a hypersensitivity reaction should be considered. Bovine collagen injection may rarely cause allergic reactions. The development of erythema, edema, induration, pruritus, and pain is a sign of hypersensitivity. Hyaluronic acid fillers generally do not require testing, if there is a hypersensitivity reaction due to them, they can be treated with hyaluronidase injection [29].

It is possible that filler application triggers herpes infections. Therefore, in patients with a history of herpes before lip augmentation, prophylactic antiviral therapy is recommended. If there is an active herpes lesion in the area to be filled, injection should be delayed until these lesions disappear completely. Post-filling infection and contamination rarely occur when the application is done under appropriate conditions. Skin and soft tissue pathogens such as *Staphylococcus aureus* are the most common causative agents. In this study, we did not face any complications in the study population [30].

The limitations of the study include the fact that it was performed retrospectively and that computer-assisted photographic and objective measurements were not performed.

## Conclusions

The migration of lip filler to the cutaneous upper lip is influenced by multiple factors, including the type of filler, injection technique, and patient anatomy. When specifying injection techniques, it cannot be ignored that the direction of the needle is one of the factors that must be evaluated. Understanding these factors and adopting tailored approaches to filler injections can significantly improve aesthetic outcomes and advance the field of medical aesthetics field.

In this study, patient satisfaction was the highest in the group in which the needle was orientated from top to bottom considering the migration to the cutaneous part of the upper lip. These findings showed that the direction of the needle during injection also determines the direction of distribution of the filler in the lip and may be an important factor in patient satisfaction.
